# Effect of Graphene Oxide Nanosheets on Physical Properties of Ultra-High-Performance Concrete with High Volume Supplementary Cementitious Materials

**DOI:** 10.3390/ma13081929

**Published:** 2020-04-19

**Authors:** Yu-You Wu, Jing Zhang, Changjiang Liu, Zhoulian Zheng, Paul Lambert

**Affiliations:** 1 School of Transportation, Civil Engineering and Architecture, Foshan University, Foshan, Guangdong 528000, China; 2 College of Mechanical Engineering, Dongguan University of Technology, Donghuan, Guangdong 523808, China; 3 School of Civil Engineering, Guangzhou University, Guangzhou, Guangdong 51000, China; 4 School of Civil Engineering, Chongqing University, Chongqing 40000, China; zhengzl@cqu.edu.cn; 5 Materials and Engineering Research Institute, Sheffield Hallam University, Sheffield S1 1WB, UK

**Keywords:** graphene oxide, ultra-high-performance concrete, supplementary cementitious materials, physical properties

## Abstract

Nanomaterials have been increasingly employed for improving the mechanical properties and durability of ultra-high-performance concrete (UHPC) with high volume supplementary cementitious materials (SCMs). Recently, graphene oxide (GO) nanosheets have appeared as one of the most promising nanomaterials for enhancing the properties of cementitious composites. To date, a majority of studies have concentrated on cement pastes and mortars with fewer investigations on normal concrete, ultra-high strength concrete, and ultra-high-performance cement-based composites with a high volume of cement content. The studies of UHPC with high volume SCMs have not yet been widely investigated. This paper presents an experimental investigation into the mini slump flow and physical properties of such a UHPC containing GO nanosheets at additions from 0.00 to 0.05% by weight of cement and a water–cement ratio of 0.16. The study demonstrates that the mini slump flow gradually decreases with increasing GO nanosheet content. The results also confirm that the optimal content of GO nanosheets under standard curing and under steam curing is 0.02% and 0.04%, respectively, and the corresponding compressive and flexural strengths are significantly improved, establishing a fundamental step toward developing a cost-effective and environmentally friendly UHPC for more sustainable infrastructure.

## 1. Introduction

Nanomaterials have been increasingly employed over recent years for improving the mechanical properties and durability of ultra-high-performance concrete (UHPC) with high volume supplementary cementitious materials (SCMs) [1,2]. UHPC normally exhibits exceptional mechanical properties and excellent durability and has become one of the most significant new construction materials [3,4]. Typically, the components of UHPC consist of Portland cement, silica fume (SF), quartz sand (QS), superplasticizers (SPs), water, steel fibers (STFB), and optionally, quartz powder (QP) [5,6]. However, UHPC has not yet been widely applied in engineering practice due to its high materials cost, embedded energy and environmental impact [7]. Various measures have been developed to overcome these drawbacks and one of these is achieved by either replacing the Portland cement with high volume supplementary cementitious materials (SCMs) such as fly ash (FA), ground granulated blast furnace slag (GGBFS), silica fume (SF), rice husk ash (RHA), and metakaolin (MK), or by reducing the content of steel fibers [6,8]. It is important to note that the measures performed should not result in significantly decreasing the mechanical properties and durability of UHPC [9,10,11], which is highly challenging. 

Recently, graphene oxide (GO) has appeared as one of the most promising nanomaterials for enhancing the mechanical properties and durability of cementitious composites [12,13]. This could be related to the fact that GO is hydrophilic, and therefore is easily dispersible in water compared to other carbon-based nanomaterials such as graphene nanoplatelets (GNP) or carbon nanotubes (CNTs) [14]. As such, it can cost-effectively enhance the properties of UHPC. Pan et al. [15] reported that the compressive strength and flexural strength of ordinary Portland cement pastes with the addition of 0.05% GO nanosheets (by weight of cement) were increased by 15–33% and 41–58%, respectively, potentially associated with enhanced mechanical interlocking, interaction between the microcracks and GO nanosheets, promotion of the hydration process and the formation of powerful interfacial forces between carboxylic groups and hydration products. Lv et al. [16] observed that when the content of GO nanosheets was 0.05% (by weight of cement), the compressive strength, flexural strength and tensile strength of ordinary Portland cement mortars were increased by 38.9%, 60.7%, and 78.6%, respectively, indicating that GO nanosheets played a critical role in enhancing the strength of such mortars. Mohammed et al. [17,18,19] reported that the addition of GO nanosheets can enhance the mortar resistance to chloride penetration, carbonation, and freeze–thaw cycling. 

Compared to GO nanosheet applications in pastes and mortars, it was employed somewhat later for improving the properties of concrete. Wu et al. [20] reported that the compressive strength, flexural strength and split tensile strength of normal concrete (NC) were enhanced by an increase at the level of GO nanosheets from 0.02% to 0.08%, whereas the slump of concrete decreases with increasing GO nanosheet content. Moreover, the authors also found that 0.03% is the optimum value of GO nanosheet dosage for improving the split tensile strength of the concrete specimens with a water–cement ratio of 0.5. Devi et al. [21] observed that the sorptivity and permeability of concrete with GO reduced with increasing GO content from 0.02% to 0.08% compared to control concrete mixes. Lu et al. [22] found that the fluidity of ultra-high strength concrete (UHSC) decreased with increasing addition of GO nanosheets while the researchers also reported that the addition of GO nanosheets improved the flexural and compressive strengths of UHSC, whereas the addition of GO nanosheets significantly increased the deformation ability of UHSC. It should be noted that other factors such as mineralogy and microstructure of the coarse aggregates might also affect the final strength of the concrete specimens [23]. Ren [24] reported that the addition of GO improved the compressive strength and flexural strength of self-compacting high performance cement-based composite and its resistance to chloride ion penetration and erosion with high cement dosage, while its fluidity decreased. However, there is insufficient information on the effect of GO on the properties of UHPC with high volume SCMs. It is therefore of interest to conduct research to bridge this gap and help the development of cost-effective and eco-efficient UHPC with enhanced properties.

In this study, the effects of GO on the mini slump flow and physical properties of UHCP with high volume SCMs are investigated experimentally under standard and steam curing. The GO nanosheet content is at additions of 0.00%, 0.01%, 0.02%, and 0.03% by weight of cement, with a water–cement ratio of 0.16. The physical properties evaluated include the compressive strength and flexural strength. Additionally, a working mechanism for improving the physical properties of UHPC with high volume SCMs by the addition of GO nanosheets is also discussed.

## 2. Materials and Methods

### 2.1. Materials

Ordinary Portland cement type 42.5 (OPC 42.5) complying with GB/T175-2007 [25], class F FA, GGBFS, and SF was used as binder materials for all UHPC mixes. The chemical compositions and physical properties of the cementitious materials are summarized in Table 1. The diameter range of the SF is 100–300 nm and its BET surface area is 19,500 m^2^/kg. The Blaine surface area of FA and GGBFS is 330 m^2/^kg and 418 m^2/^kg, respectively.

QS was employed and was composed of three grades with a diameter range of 0.212 to 0.425 mm (QS1), 0.425 to 0.85 mm (QS2) and 0.85 to 2.00 mm (QS3), respectively. Copper-coated straight micro STFB with a 12–16 mm length and 0.18–0.25 mm diameter and a tensile strength of 2800 MPa was employed. A polycarboxylate-based superplasticizer (PCs) with a water-reducing capacity greater than 30% was used to improve the workability of UHPC.

**Figure 1:**
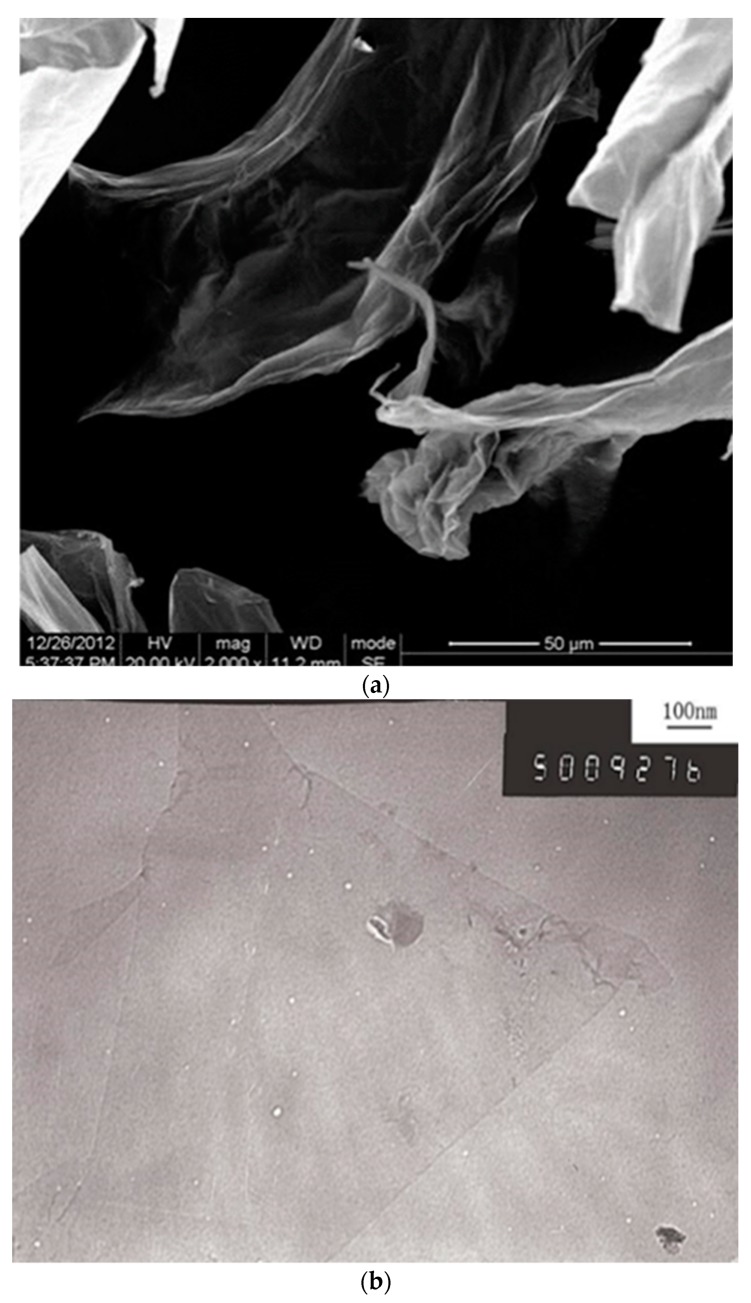
Images of GO nanosheets. (**a**) SEM image of multilayer GO. (**b**) TEM image of GO.

GO nanosheets were used as a water dispersion solution which was synthesized by using a modified Hummers method [26] at Chengdu Institute of Organic Chemistry, China. The main parameters of the GO nanosheet are shown in Table 2. Figure 1a shows a scanning electron microscope (SEM) image of multilayer GO nanosheets. Figure 1b shows a transmission electron microscopy (TEM) image of a typical GO nanosheet with wrinkled and folded features.

### 2.2. UHPC Specimens Preparation

Six concrete mixes, designated as UHPC0, UHPC1, UHPC2, UHPC3, UHPC4, and UHPC5, were prepared containing 0%, 0.01%, 0.02%, 0.03%, 0.04%, and 0.05% of GO nanosheets by weight of cement, respectively. The UHPC mixture proportions are shown in Table 3, based on an earlier study [27]. The mixing water incorporated the GO nanosheets as a dispersion. All mixes had a water-to-binder ratio (W-B) of 0.16 and a sand-to-binder ratio (S-B) of 1.0. The mass fraction of OPC, SF, FA, and GGBFS was 42%, 17%, 33%, and 9% of total binder, respectively. The mass fraction of QS1, QS2, and QS3 was 43%, 31%, and 25% of total QS. The content of PCs was 0.4% by weight of binder. The dosage of STFB was 1.5% by volume of concrete.

The mixing procedure consisted of five steps: (1) the potable water was pre-mixed with the GO nanosheet water dispersion in a separate vessel and stirred at 2000 rpm for five minutes; (2) dry QS composed of QS1, QS2, and QS3 was mixed for one minute in a vertical shaft mortar mixer and then dry cementitious materials and PCs powder were added prior to mixing at 60rpm for a further one minute; (3) the pre-mixed solution was added and mixed for two minutes; (4) STFB was gradually added within one minutes at 60 rpm; (5) the final mixing was performed at 60rpm for three minutes. The fresh UHPC mixture was cast into pre-oiled molds and compacted on an electric vibration table after which the specimens were covered with a polyethylene sheet and cured in the laboratory for 24 h.

### 2.3. Curing Regimes

**Figure 2:**
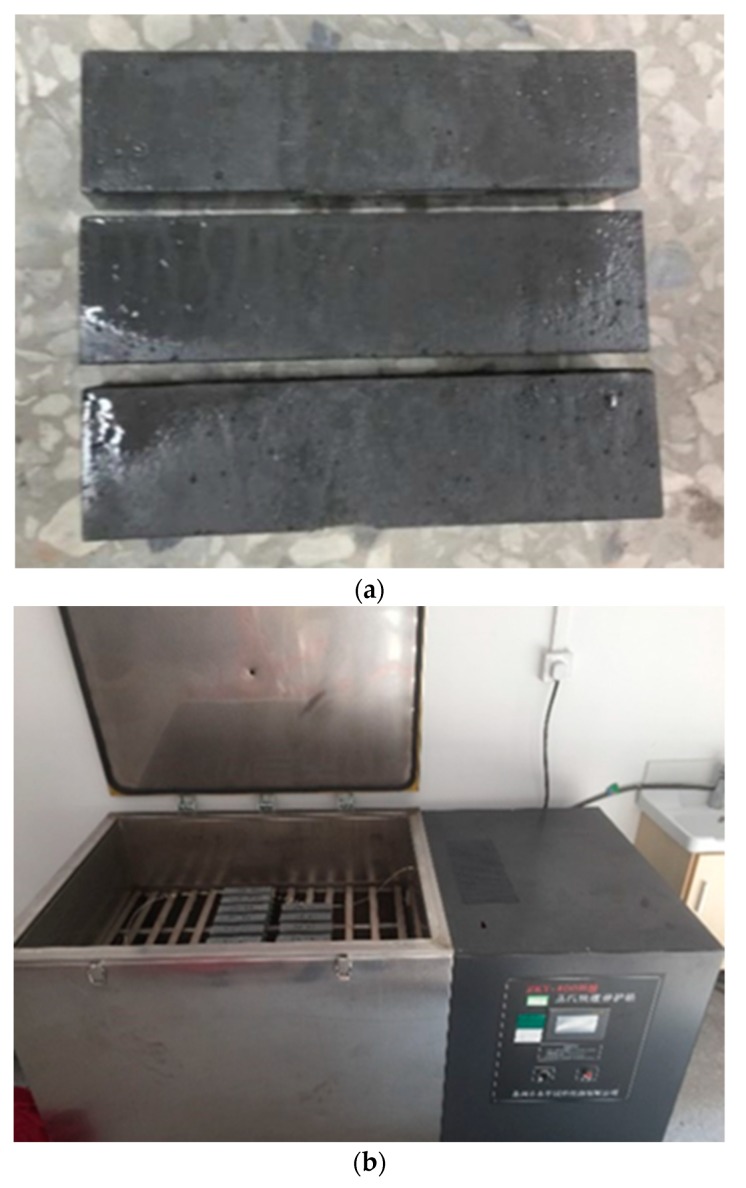
(**a**) Specimens for flexural strength test. (**b**) Specimens cured in a steam box.

Following demolding, the specimens were divided into two groups. The specimens of Group One were cured at standard room temperature and high relative humidity, designated “standard curing”. The specimens of Group Two were first cured under a steam box and then followed by the standard curing, designated “steam curing”. For standard curing, the specimens were cured at a relative humidity greater than 95% and a temperature of 21 ± 2 °C until the time of testing. For steam curing, the specimens were moved into a steam box for curing as shown in Figure 2, where the temperature was elevated at a rate of 10 °C per hour to 90 °C and then stayed at 90 °C for 24 h, which was followed by lowering the temperature at a rate of 10 °C per hour to the room temperature and then cured at a relative humidity greater than 95% and a temperature of 21 ± 2 °C until tested age in this study.

### 2.4. Experimental Methods

#### 2.4.1. Fluidity Test

The fluidity of the fresh UHPC mixture was assessed by measuring its mini slump flow. The test of mini slump flow was performed in accordance with Chinese Standard GB/T2419–2005 [28].

#### 2.4.2. Compressive and Flexural Strength Tests

**Figure 3:**
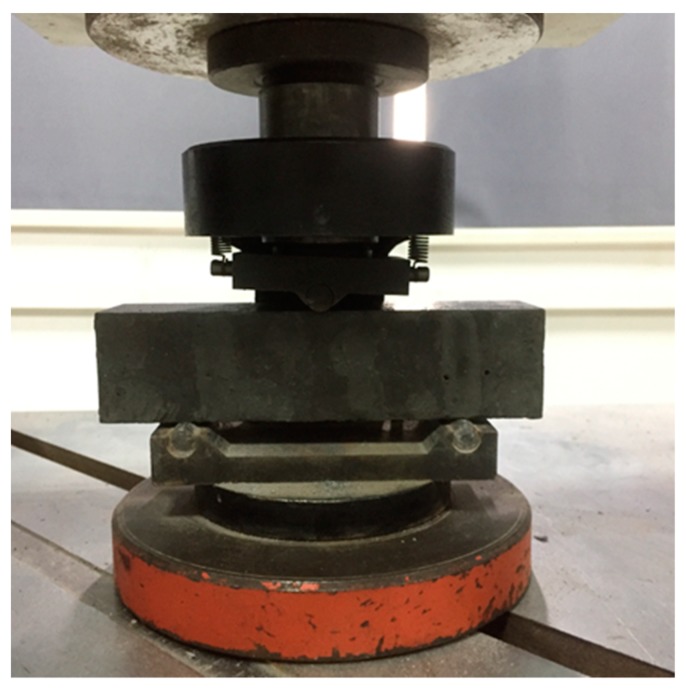
Test set-up for flexural strength test.

The compressive and flexural strengths of specimens were measured at 7 and 28 days. The specimens were tested in accordance with Chinese Standard GB/T31387–2015 [29]. The compressive strength test of specimens with dimensions of 40 × 40 × 40 mm and the flexural strength test of specimens with dimensions of 40 × 40 × 160 mm, as shown Figure 3, were carried out using MTS testing equipment. The loading rates employed were 1.2 MPa/s and 0.12 MPa/s, respectively. Each compressive strength and flexural strength value presented in this study is the average of six test results.

## 3. Results and Discussion

### 3.1. Effect of GO Nanosheets on Fluidity of UHPC Mixture with High Volume SCMs

**Figure 4:**
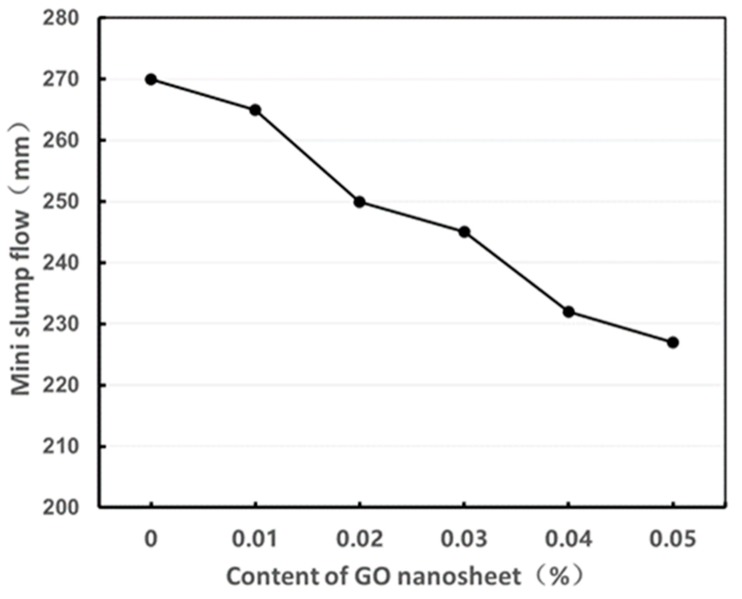
Mini slump flow of UHPC with different GO nanosheet contents.

The results obtained from the mini slump flow test are shown graphically in Figure 4. Each value presented is the average of two test results. It is observed that the values of mini slump flow decreased with the increase of GO nanosheet dosage. The values for the fresh UHPC mixes with GO nanosheet contents of 0.0%, 0.01%, 0.02%, 0.03%, 0.04%, and 0.05% are 270 mm, 265 mm, 250 mm, 245 mm, 232 mm, and 227mm, representing a decreasing rate of 1.9%,7.4%,9.3%,14.1%, and 15.9%, respectively, when compared to the reference mix UHPC0. This was attributed to the decrease in the availability of water in the fresh UHPC mix from wetting due to the high specific surface area of GO nanosheet [15]. The results have demonstrated that the addition of GO nanosheet reduced the flowability of UHPC mixture with high volume SCMs and thus its workability. Similar results were also reported in previous studies on NC [20,21], UHSC [22], self-compacting ultra-high-performance cement-based composites with high cement content [24], all containing GO nanosheets. Elsewhere, a previous study regarding UHPC enhanced with the graphite nanoplatelet (GNP), which is another type of graphene-based nanosheet (GNS), indicated that when GNP content was less than 0.05%, the flowability of the UHPC increased, whereas it decreased when the GNP content was greater than 0.05%, suggesting the flowability of the UHPC is affected by the dosages of both the high range water reducer and the GNP [30]. Therefore, further investigation in present study is required.

### 3.2. Effect of GO Nanosheets on Compressive and Flexural Strengths of UHPC with High Volume SCMs

**Figure 5:**
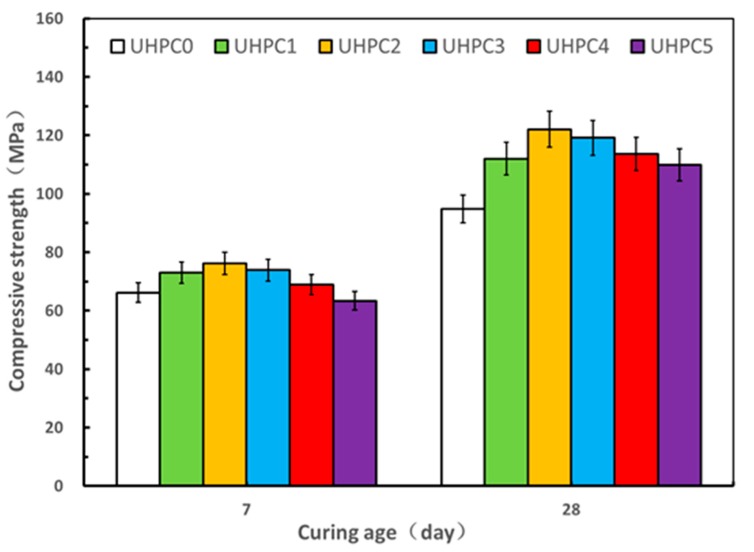
Effect of GO nanosheets on compressive strength under standard curing.

**Figure 6:**
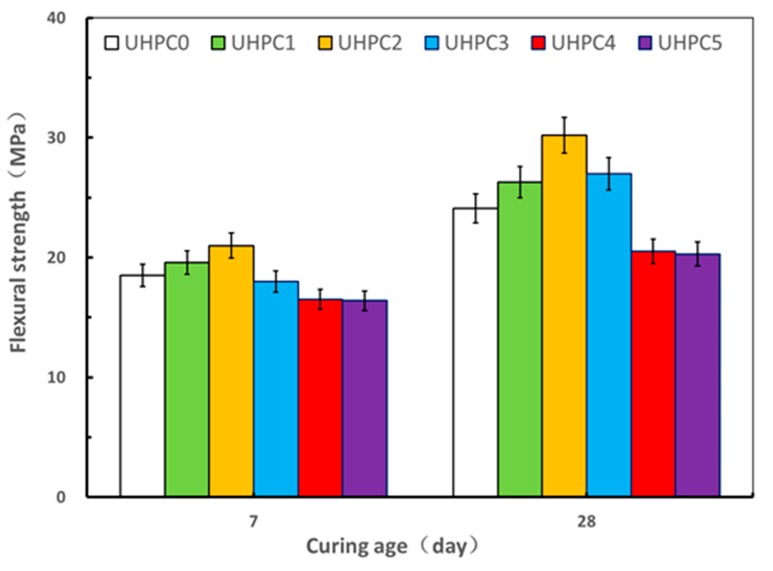
Effect of GO nanosheets on flexural under standard curing.

The results of the compressive and flexural strength tests on the UHPC specimens with the varying contents of GO nanosheets under standard curing at ages of 7 and 28 days are shown in Figure 5 and Figure 6. It is apparent that both the compressive strength and flexural strength of the UHPC improves as the dosage of GO nanosheets is increased from 0.00% to 0.02%, whereas the values decreased with additions of GO nanosheets from 0.03% to 0.05% at both ages of 7 and 28 days. It therefore demonstrated that an optimal dosage of GO nanosheets for the UHPC with the high volume SCMs under standard curing was in the order of 0.02%, where the corresponding value of mini slump flow was 250 mm. With the GO nanosheet addition at 0.02%, the value of the compressive strength and the flexural strength of UHPC2 at 28 days was 122.1 MPa and 30.2 MPa, which represented an increase of 28.7% and 25.3%, respectively, compared to the reference specimens (UHPC0). This can be associated with the “nano size effect” of GO nanosheets accelerating the hydration reactions of the cementitious materials to improve the microstructure of UHPC and the “bridging effect” of GO nanosheets for controlling microcracks [30]. It can be also seen that both compressive and flexural strengths at age of 28 days were significantly higher than those at age of 7 days.

However, as referred to previously, both the compressive strength and flexural strength of the UHPC specimens decreased when the content of GO nanosheets increased from 0.03% to 0.05%, where the corresponding compressive strength at age of 28 day decreased from 122.1 MPa to 110.0 MPa, while the corresponding flexural strength decreased from 30.2 MPa to 20.3 MPa. Furthermore, the flexural strengths of UHPC4 and UHPC5 were comparable and both were even less than the flexural strength of the reference specimen UHPC0 at ages of 7 days and 28 days. This could be attributed to the higher dosage of GO nanosheets that might not be uniformly dispersed [13]. On the other hand, well-dispersed GO nanosheets can more effectively promote the formation of cement hydration products, better inhibit the formation of large-size pores, and optimize the pore size distribution compared to when GO nanosheets agglomerate [31]. The results in present study demonstrate that the addition of the optimal GO nanosheet dosage can improve the compressive strength and flexural strength of UHPC with high volume SCMs, leading to the development of a low cost and environmentally friendly UHPC under standard curing.

**Figure 7:**
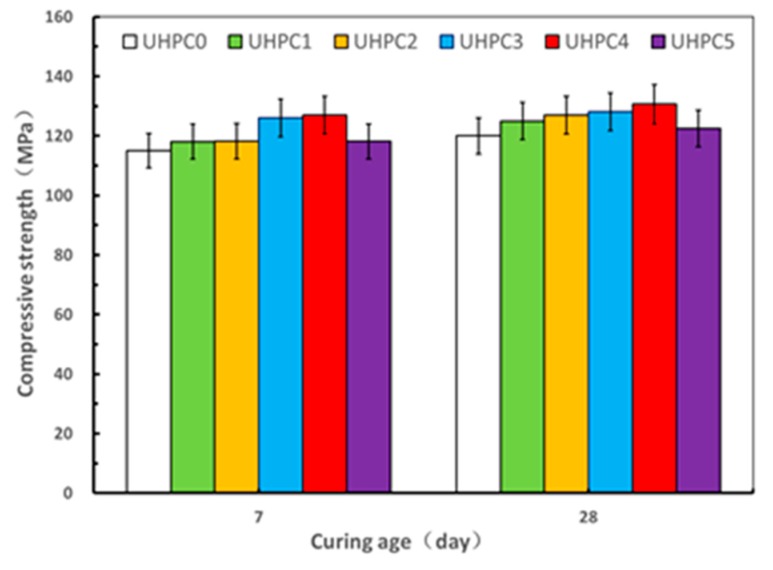
Effect of GO nanosheets on compressive strength under steam curing.

**Figure 8:**
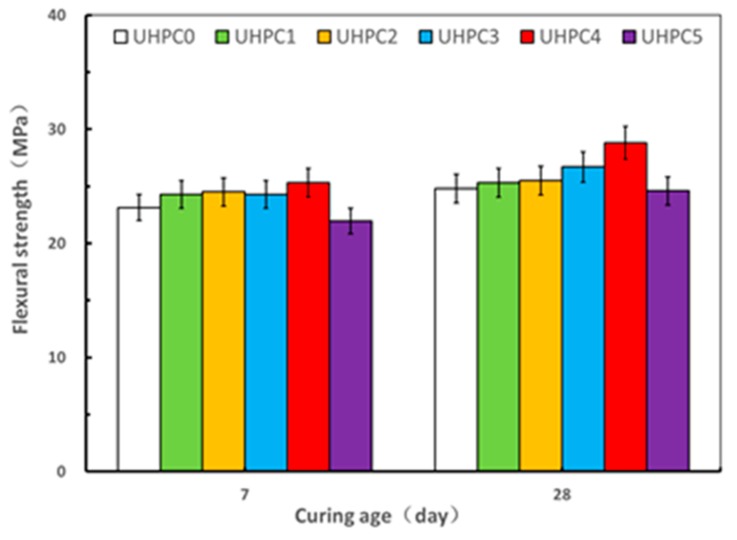
Effect of GO nanosheets on flexural under steam curing.

The results of the compressive and flexural strength tests on the UHPC specimens with varying contents of GO nanosheets under steam curing at ages of 7 and 28 days are shown in Figure 7 and Figure 8. It can be seen that the compressive strength and flexural strength of the UHPC increased when the dosage of GO nanosheets was increased from 0.00% to 0.04%, whereas such strengths of UHPC specimens decreased when the GO nanosheets content was 0.05% at ages of 7 and 28 days, indicating that an optimal dosage of GO nanosheets for the UHPC with the high volume SCMs under steam curing was in the order of 0.04%, where the corresponding value of mini slump flow was 232.00 mm. With the GO nanosheet content of 0.04%, the value of the compressive strength and the flexural strength of UHPC4 at 28 days was 130.6 MPa and 28.8 MPa, which represented an increase of 8.8% and 16.1%, respectively, compared to the reference specimens (UHPC0). This can be associated with the both “nano size effect” and “bridging effect” of GO nanosheets [30]. As discussed above, the compressive strength and flexural strength of UHPC5 specimens were less than those of UHPC 4 at both ages of 7 and 28 days. For example, the compressive strength and flexural strength of UHPC5 at 28 days were 122.4 MPa and 24.6 MPa, respectively, representing a decrease of 6.2% and 14.5% compared to the UHPC4. Additionally, the flexural strengths of UHPC5 were less than that of the reference specimen UHPC0 at both ages of 7 and 28 days. This may be due to the high content of GO nanosheets that could not be adequately dispersed [13]. On the other hand, well dispersed GO nanosheets can more effectively promote the formation of cement hydration products, better inhibit the formation of large-size pores and optimize the pore size distribution compared to when GO nanosheets agglomerate [31]. The results from present study indicates that the compressive strength and flexural strength of UHPC with high volume SCMs can be enhanced by ensuring an optimal GO nanosheet content, therefore aiding the development of a low cost and environment friendly UHPC under steaming curing.

Comparing the results obtained from the specimens under the different curing regime, it is apparent that the optimal dosage value of GO nanosheets for improving the compressive and flexural strengths of specimens at standard curing and under steam curing were in the order of 0.02% and 0.04% by weight of cement, respectively, and the corresponding values at 28 days were 122.4 MPa and 130.6 MPa. This suggests that the curing regime is one of the key factors to affect the optimal dosage of GO nanosheets for UHPC with high volume SCMs for a given mix. This optimal dosage range is different from that observed for the UHSC incorporating GO nanosheets under standard curing, where it was indicated that the optimal dosage value was 0.01% [22] while that value obtained by a previous study on the effect of GO nanosheets on a self-compacting ultra-high performance cement-based composite with high volume cement content was 0.03% at standard curing [24]. Therefore, the optimal value of GO nanosheets is also affected by other factors such as water-to-cement ratio, raw material type, SCMs, etc. Moreover, it is observed that the compressive strength of specimens with the same content of GO nanosheets under steam curing were generally greater than those with standard curing for a given age. This was attributed to the effect of the steam curing that promotes the pozzolanic reactivity between calcium hydroxide from the cement hydration and SCMs to improve the microstructure [32]. Additionally, by prolonging the curing time, it should be possible for mixes with standard curing to reach a higher compressive strength, comparable to those obtained under steam curing [33]. It should be noted that for the flexural strength, the trend was slightly different from the compressive strength. The flexural strength values of specimen with the GO nanosheet dosages of 0.01% and 0.02% were 25.3 MPa and 25.5 MPa at 28 days under steam curing, respectively. Both were less than the corresponding values of 26.3 MPa and 30.2 MPa at age of 28 days under standard curing, which highlights the need for further research. It should be noted that the maximum value of compressive strength at standard curing and under steam curing at 28 days were 122.4 MPa and 130.6 MPa, respectively, and both may be further improved by either optimizing particle size distribution or lowering W/B ratio. Further investigation is therefore recommended.

### 3.3. Analysis and Comparison of Mechanical Properties

Thanks to their extraordinary mechanical, chemical, and thermal properties, graphene-based nanosheets (GNS), such as graphene nanosheets, GO nanosheets, and graphite nanoplatelets (GNP), have been used to improve the mechanical properties and durability of concrete over recent years [13]. Based on the current literature and the results from this study, some mechanical properties of NC, UHSC, and UHPC with the addition of graphene nanosheets, GO nanosheets, and GNP are summarized in Table 4. Standard curing was employed for specimens in previous studies [20,21,22,24,34] and UHPC2 in present study while specimens prepared by Wang et al. [35] were cured in 25 °C water for 3 months and followed by the air curing (room temperature) for 7 days. Steam curing was used for UHPC4 (see Table 3) in the present study. The results reported by Lu et al. [22] and Wang et al. [35] were obtained at 7 and 97 days, respectively, and the remaining results were obtained at 28 days.

It can be seen that both compressive strength and flexural strength for all types of concrete incorporating graphene nanosheets, GO nanosheets, and GNP were enhanced while the tensile strength (TS) was improved, where such results were available. This can be associated with the both nano size (or nucleation) effect and bridging effect of these nanosheets, with the former promoting the hydration reactions of the cementitious materials for improving the microstructure of concrete and the latter preventing the expansion of the microcracks through concrete matrix [13,34]. For the UHPC with STFB, such as the results given by Ren [24] and Meng et al. [30] in addition to this study, the increasing rate of the flexural strength was significantly greater than that of the compressive strength for a given addition of GO nanosheets or GNP, which might be attributed to the improvement of the interface transition zone and the stronger bond between the concrete matrix and STFB [30]. However, further investigation may be required to confirm these proposed affects. For UHPC containing graphene nanosheets without STFB, such as the results obtained by Wang et al. [35], it is apparent that the compressive toughness (CT), dynamic compressive strength (DCS), dynamic peak strain (DPS), dynamic ultimate strain (DUS), and impact toughness (IT) were significantly improved compared with the compressive strength and flexural strength. Except for the nucleation effect and bridging effect of graphene nanosheets, it was also associated with the interlaminar slip and structural fracture of graphene nanosheet plus further absorbed strain energy released by cracking, therefore improving the mechanical properties of the UHPC.

As analyzed above, it is verified that the addition of GO nanosheets can improve the mechanical properties of NC, UHSC, and UHPC with high volume cement, and UHPC with high volume SCMs. It is also demonstrated that the results from the present study lead to the development of a low cost and environmentally friendly UHPC for a more sustainable infrastructure. Further work regarding UHPC with high volume SCMs is currently underway and the results will be reported once successfully completed.

## 4. Conclusions

In the present study on the effect of GO nanosheets on the physical properties of UHPC with high volume SCMs we draw the following conclusions.

(1) The mini slump flow of UHPC with high volume SCMs decreases when the content of GO nanosheets increases from 0.01% to 0.05% by weight of cement for a given water to binder ratio of 0.16.
(2) The optimal dosage value of GO nanosheets for improving the compressive and flexural strengths of UHPC with high volume SCMs at standard curing is of the order of 0.02% by weight of cement while it is of the order of 0.04% under steam curing at 28 days, suggesting the curing regime is one of the key factors to affect the optimal dosage of GO nanosheets for such UHPC for a given mix.
(3) The compressive strength and flexural strength of UHPC with high volume SCMs and containing the optimal GO dosage of 0.02% at standard curing at 28 days are 122.4 MPa and 30.2 MPa, respectively, whereas the compressive strength and flexural strength of such UHPC containing the optimal GO dosage of 0.04% under steam curing at 28 days are 130.6 MPa and 28.8 MPa, respectively, demonstrating that the addition of the optimal GO nanosheet dosage can improve the strengths of UHPC with high volume SCMs. The results set up a fundamental step toward developing a low cost and environmentally friendly UHPC for more sustainable infrastructure.
(4) The improvement of mechanical properties may be associated with the nano size effect, nucleation effect and bridging effect of GO nanosheets. Additionally, the interlaminar slip and structural fracture of GO nanosheets may further absorb strain energy released by cracking, thereby improving the mechanical properties. This is the subject of further research.

## Figures and Tables

**Table 1:** Chemical composition and physical properties of cementitious materials *.

Materials		OPC	SF	FA	GGBFS
SiO_2_ (wt%)		21.00	95.30	53.97	42.00
Al_2_O_3_ (wt%)		5.40	1.20	31.15	16.00
Fe_2_O_3_ (wt%)		2.80	0.90	4.16	-
CaO (wt%)		64.68	0.40	4.01	40.00
MgO (wt%)		1.40	0.80	1.01	-
SO_3_ (wt%)		2.19	-	0.73	1.72
K_2_O (wt%)			-	2.04	-
Na_2_O (wt%)			1.10	0.89	-
TiO_2_ (wt%)			-	1.13	-
P_2_O_5_ (wt%)			-	0.67	-
Cl (wt%)		0.01	-	0.13	0.05
NiO (wt%)			-	0.11	-
Loss on ignition (g)		2.52	0.30	-	0.23
Blaine surface area (m^2/^kg)		357	-	330	418
BET surface area (m^2/^kg)		-	19,500	-	-
Specific gravity (kg/m^3)^		3140	2200	2280	2900
Setting time (min)	Initial	203	-	-	-
	Final	250	-	-	-
Compressive strength (MPa)	3d	27.40	-	-	-
Flexural strength (MPa)	3d	5.90	-	-	-

* OPC: ordinary Portland cement; SF: silca fume; FA: fly ash; GGBFS: ground granulated blast furnace slag.

**Table 2:** The composition and dimensions of graphene oxide nanosheet.

Items	Carbon (%)	Oxygen (%)	Length/Width (μm)	Thickness (nm)
Value range	45–60	40–55	1.0–5.0	0.6–1.5

**Table 3:** Ultra-high-performance concrete (UHPC) mixture proportions (kg/m^3^) *.

Mix.	Water	Cement	SF	FA	GGBFS	QS1	QS2	QS3	GO	PCs	STFB
UHPC0	151	453.80	180	360	93	473	341	274	0.00	4.35	120
UHPC1	151	453.75	180	360	93	473	341	274	0.05	4.35	120
UHPC2	151	453.71	180	360	93	473	341	274	0.09	4.35	120
UHPC3	151	453.66	180	360	93	473	341	274	0.14	4.35	120
UHPC4	151	453.62	180	360	93	473	341	274	0.18	4.35	120
UHPC5	151	453.57	180	360	93	473	341	274	0.23	4.35	120

* SF: silca fume; FA: fly ash; GGBFS: ground granulated blast furnace slag; QS: quartz sand; GO: graphene oxide; PCs: polycarboxylate-based superplasticizer; STFB: steel fiber.

**Table 4:** Comparison of physical properties *.

References	Matrix	GNS Type	GNS Content (%)	W/B	Performance Improvement
Wu et al. [20]	NC	GO	0.03	0.5	15.2% CS, 3.8% FS
	NC	GO	0.04	0.5	21.8% CS, 8.1% FS
Devi et al. [21]	NC	GO	0.04	0.45	10.0% CS, 8.0% TS
	NC	GO	0.06	0.45	13.0% CS, 12.0% TS
Chen et al. [34]	NC	GO	0.02	0.35	4.0% CS, 3.8% FS
	NC	GO	0.05	0.35	8.0% CS, 4.7% FS
Lu et al. [22]	UHSC	GO	0.01	0.2	3.6% CS, 11.8% FS
	UHSC	GO	0.03	0.2	4.5% CS, 6.9% FS
Meng et al. [30]	UHPC	GNP1	0.3	0.2	6.6% CS, 59.2% FS, 45.5% TS
	UHPC	GNP2	0.3	0.2	5.7% CS, 38.9% FS, 39.8% TS
Wang et al. [35]	UHPC	Graphene	0.5	0.19	10.2% CS, 1.4% FS, 40.2% CT, 63.9% DCS, 66.0% DPS, 32.7% DUS, 117% IT
Ren [24]	UHPC	GO	0.03	0.22	14.5% CS, 21.2% FS
	UHPC	GO	0.05	0.22	14.1% CS, 22.0% FS
Present study	UHPC2	GO	0.02	0.16	28.8% CS, 25.3% FS
	UHPC4	GO	0.04	0.16	8.8% CS, 16.1% FS

* GNP1: GNP with a specific surface area of 300 m^2^/g; GNP2: GNP with a specific surface area of 150 m^2^/g; CS: compressive strength; FS: flexural strength; TS: tensile strength; CT: compressive toughness; DCS: Dynamic compressive strength; DPS: dynamic peak strain; DUS: dynamic ultimate strain; IT: impact toughness.
